# A retrospective analysis of adverse drug reaction case reports for PARP inhibitors: a comparative study of olaparib and niraparib

**DOI:** 10.3389/fphar.2026.1935594

**Published:** 2026-09-24

**Authors:** Dong Zou, Jiaqi Yao, Zhaoyan Zang

**Affiliations:** 1 Department of Pharmacy, Sir Run Run Shaw Hospital, College of Medicine, Zhejiang University, Hangzhou, China; 2 Sir Run Run Shaw Hospital, College of Medicine, Zhejiang University, Hangzhou, China; 3 Department of Pharmacy, Ningbo NO.2 Hospital, Ningbo, Zhejiang, China

**Keywords:** ADRs, case report, niraparib, olaparib, PARP inhibitors, systematic analysis

## Abstract

**Background:**

Olaparib and niraparib are the most widely used poly (ADP-ribose) polymerase inhibitors (PARPis) in clinical practice. However, clinical trial data inadequately capture rare and delayed adverse drug reactions (ADRs) in real-world settings, and a systematic comparison of their ADR profiles is lacking, hindering individualized therapy and risk management. This study systematically analyzes published case reports of ADRs associated with these two drugs to summarize their characteristics.

**Methods:**

This retrospective study systematically retrieved case reports on olaparib- and niraparib-related ADRs from seven databases (CNKI, Wanfang, VIP, PubMed, Web of Science, ScienceDirect, and Embase) up to June 2026. Following screening, we extracted baseline data, ADR manifestations, interventions, and outcomes. Adverse events were classified by System Organ Class using the Medical Dictionary for Regulatory Activities (MedDRA), and comparative analyses were conducted on ADR spectra, time-to-onset, and prognosis between the two drugs.

**Results:**

A total of 45 case reports involving 55 patients (68 ADR events) were included, of which 33 cases (40 events) were olaparib-related and 22 cases (28 events) niraparib-related. Seven patients were male (all olaparib users) and 48 females (male-to-female ratio 1:6.8). Most patients were aged >50 years (47 cases, 85.45%), with a mean age of 61.02 ± 10.76 years. Ovarian cancer (OC) was the predominant primary disease (40 cases, 72.73%). Both drugs exhibited a predilection for middle-aged and elderly patients, with the first 30 days of treatment representing the peak period for ADR occurrence; with toxicities affecting the haematological, cutaneous, and respiratory systems in both groups. Distinct toxicity profiles were also observed: olaparib-associated ADRs were multisystemic, involving skin and subcutaneous tissue disorders (13 events, 32.50%), respiratory and mediastinal disorders (11 events, 27.50%), haematological and lymphatic disorders (9 events, 22.50%), and hepatobiliary disorders (4 events, 10.00%), with delayed organ toxicities such as drug-induced liver injury and myelodysplastic syndrome. Temporally, olaparib exhibited a bimodal onset pattern: early cutaneous toxicity followed by mid-to-late pulmonary and hepatobiliary injury. Niraparib-associated ADRs were highly concentrated in the haematological system (20 events, 71.43%), with thrombocytopenia as the core feature, accompanied by neuromuscular toxicities including dysgeusia and elevated creatine kinase; temporally, an early unimodal distribution was observed, with new ADRs significantly reduced after day 91. Twenty-four patients in the olaparib group improved after drug discontinuation, dose adjustment, or symptomatic treatment, seven died due to ADRs or disease progression, and 2 had unreported outcomes; in the niraparib group, 21 improved, 1 showed no improvement, and no drug-related deaths occurred. Upon rechallenge, ADRs recurred mostly within 1 week, and dose reduction effectively lowered recurrence risk. Causality assessment using the Naranjo scale identified 8 cases as “definite”, 42 as “probable”, and 5 as “possible”.

**Conclusions:**

Olaparib and niraparib presented markedly different ADR profiles. Drug selection and monitoring should be individualized based on patient characteristics in clinical practice. Olaparib requires sustained vigilance for cutaneous, pulmonary, and hepatobiliary toxicities, with attention to delayed organ injury; niraparib warrants prioritized prevention of early thrombocytopenia. These findings complement real-world safety evidence for PARP inhibitors and provide a reference for individualized drug selection and stratified safety monitoring in clinical practice.

## 1 Introduction

PARPis occupy a pivotal role in the maintenance therapy of malignancies with homologous recombination repair deficiency (HRD), most commonly caused by germline or somatic *BRCA1/2* mutations, including ovarian, breast, and prostate cancers, by inhibiting PARP-mediated base excision repair and inducing synthetic lethality in HRD tumor cells (Bryant et al., 2005; Lord and Ashworth, 2017). To date, six PARPis have been approved globally for clinical use, namely olaparib, niraparib, talazoparib, rucaparib, fuzuloparib, and pamiparib. Among these, olaparib and niraparib, granted United States Food and Drug Administration (FDA) approval in 2014 and 2017, respectively, are the two most widely applied agents in current clinical practice. Despite sharing analogous mechanisms of action, olaparib and niraparib differ markedly in chemical structure and *in vivo* exposure, resulting in heterogeneous real-world safety profiles, including differences in ADR spectra, severity, prognosis, and rechallenge risks (Ren et al., 2024). With the expanding scope of clinical indications, individual case reports of rare and severe ADRs associated with both agents have been accumulating annually. Clinical trial data, derived from highly selected populations, cannot fully capture the spectrum of rare or delayed-onset adverse events observed in real-world clinical practice (Friedlander et al., 2023). Moreover, current literature reviews (Huang et al., 2024) predominantly focus on the safety profiles of individual agents, lacking a systematic quantitative comparison of case reports specifically between olaparib and niraparib. Consequently, the differential patterns of organ-specific toxicity distribution, clinical outcomes, and recurrence risks following drug rechallenge remain poorly defined, precluding sufficient evidence-based support for individualized monitoring, intervention, and drug selection. Noteworthy, real-world safety evidence differs in hierarchy. Large databases (such as FAERS) and EMR cohorts enable incidence calculation and risk comparison but lack granular clinical details of rare ADRs. By contrast, systematic analysis of case reports characterizes rare toxicity phenotypes in depth, yet cannot calculate incidence or quantify relative risk due to absence of exposure denominators. Specifically, all ADR proportional data in this study only reflect the composition of included case reports, and cannot represent the real-world incidence of each adverse event, nor be interpreted as common side effects of the drugs. This study design is descriptive and hypothesis-generating, with inherent limitations in causal inference and risk quantification.

This study utilizes published individual case reports of ADRs attributed to olaparib and niraparib as research materials. Employing a retrospective observational design, we systematically collected and aggregated case-level information on adverse events for both drugs. We performed a comparative analysis of the distribution characteristics of ADRs across organ systems, clinical interventions, disease outcomes, and recurrence differences after rechallenge between the two groups. By delineating the clinical phenotypes, temporal onset patterns, and prognostic characteristics of adverse events associated with these two PARPis, this study aims to complement real-world safety evidence with case-level clinical details, and provide a descriptive reference for the early recognition, stratified safety monitoring, rational dose adjustment, and individualized risk management of ADRs in clinical practice.

## 2 Methods

### 2.1 Source and search strategy

We systematically searched seven databases (CNKI, Wanfang Data, VIP, PubMed, Web of Science, ScienceDirect, and Embase) from inception to June 2026, using a combination of subject headings and free-text terms including “olaparib”, “niraparib”, “PARP inhibitor”, “adverse reaction”, “induced”, “related”, and “case report”. Two researchers independently screened the literature and extracted data according to inclusion and exclusion criteria, and any disagreements were resolved through discussion.

### 2.2 Literature inclusion and exclusion criteria

The inclusion criteria were as follows: (1) case reports of adverse reactions induced by olaparib or niraparib, including individual case reports or case analysis; (2) relatively complete general patient data and clinical treatment information, at least encompassing patient sex, age, and the principal clinical manifestations of the ADR; (3) reports explicitly stating a correlation between the ADR and olaparib or niraparib; for those that did not mention such a correlation, the authors performed a preliminary causality assessment using the Naranjo’s ADR Probability Scale (Naranjo et al., 1981); Only cases with a Naranjo score ≥4 (rated as “possible”, “probable” or “definite”) were enrolled, while cases rated “doubtful” (score ≤ 3) were excluded; (4) articles published in either Chinese or English.

The exclusion criteria were: (1) articles not published in Chinese or English; (2) review articles and duplicate publications; (3) articles with incomplete clinical data or insufficient information to determine the causality of the ADR; (4) articles with unavailable full text, or non-peer-reviewed literature such as conference abstracts. Conference abstracts were excluded because they generally lack complete clinical details, intervention records and follow-up outcomes, which are essential for standardized data extraction and reliable causality assessment. We acknowledge that this criterion may omit important cases published only in abstract form and introduce potential selection bias, but it was adopted to guarantee the data integrity and methodological quality of the study.

### 2.3 Literature screening and data extraction

Relevant information was extracted from the literature, including patient sex, age, primary disease, diagnosis, clinical manifestations, dosage, intervention measures, time to ADR onset, combined medication, and causality assessment, and statistical analysis was performed using Excel 2019. The SOCs involved in ADRs caused by PARPis were classified using the MedDRA (version 17.0). The causality of ADRs induced by PARPis was assessed using the Naranjo’s ADR Probability Scale (Naranjo et al., 1981), and the causality was categorized as “definite”, “probable”, “possible”, or “doubtful”.

### 2.4 Risk of bias assessment of included studies

Two researchers independently assessed the methodological quality of the included literature using the Joanna Briggs Institute (JBI) Critical Appraisal Checklist for Case Reports (Ma et al., 2020) and cross-checked the results. The JBI checklist comprises 8 items, each of which is rated as “yes”, “no”, “unclear”, or “not applicable”.

### 2.5 Statistical analysis

The extracted literature data were collated using Microsoft Excel 2019 software. Categorical data were presented as frequencies (n) and proportions (%).

## 3 Results

### 3.1 Search results and case characteristics

A total of 6,896 Chinese and English articles were initially identified, and after screening, 45 case reports were included (Figure 1), comprising 12 in Chinese and 33 in English. (Alshelleh et al., 2022; Álvarez-Salafranca et al., 2026; Assaf et al., 2021; Badiner et al., 2023; Chen, 2022; Clark et al., 2023; Costa and Valença, 2022; Damerval et al., 2023; Dereme et al., 2025; Gabrail and Smith, 2020; Gou et al., 2022; Hattori et al., 2025; Iinuma et al., 2021; Ishii et al., 2025; Ishimoto et al., 2023; Jin and He, 2026; Kaitsumaru et al., 2023; Kobak, 2021; Kobayashi and Yamada, 2022; Lei et al., 2026; Li et al., 2025; Ling et al., 2024; Linn et al., 2025; Maiko et al., 2022; McLaren et al., 2021; Roxo et al., 2025; Saito et al., 2023; Sekine et al., 2023; Shammo et al., 2019; Shono et al., 2024; Si et al., 2024; Stephan and Magro, 2025; Sun, 2024; Tufoni et al., 2018; Vázquez-Barrera et al., 2025; Wang et al., 2023; Wang et al., 2021; Wei et al., 2022; Wheelden et al., 2018; Xu et al., 2022; Yao and Liu, 2023; Yuan et al., 2022; Yücel et al., 2024; Zhao and Ji, 2024; Zhu et al., 2024), of which 28 were related to olaparib and 17 to niraparib.

**FIGURE 1:**
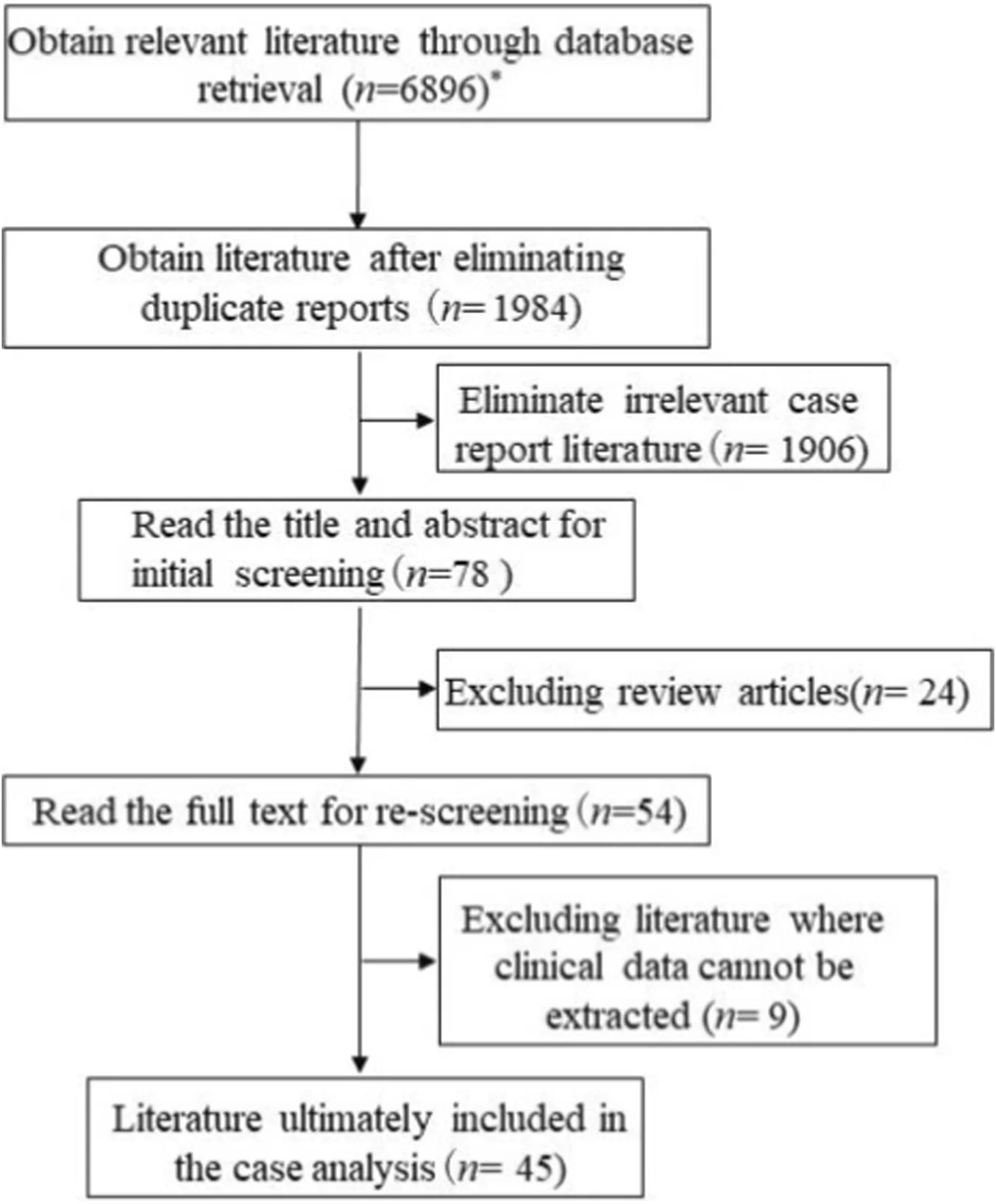
Flowchart of literature screening. Note: *The databases searched and the number of retrieved documents are as follows: PubMed (*n* = 1759), Web of Science (*n* = 2077), ScienceDirect (*n* = 2,210), CNKI (*n* = 235),VIP Date (*n* = 288), WanFang Data (*n* = 327).

### 3.2 Risk of bias assessment results of the included literature

Based on the 8 items of the JBI quality appraisal tool, the risk of bias of the included literature was assessed. All 45 included case reports met the methodological quality requirements of the JBI checklist, with no items rated as “no” or “unclear”.

### 3.3 Baseline characteristics of the patients

A total of 55 cases of ADRs to PARPis were included, comprising 33 cases associated with olaparib and 22 with niraparib. The cases originated from 14 countries: Japan (15 cases), China (15), the United States (11), Belgium (2), Turkey (2), Spain (2), and one each from Italy, Canada, the Philippines, Brazil, Switzerland, the United Kingdom, France, and Portugal. Altogether, 68 ADR events were recorded, 13 of which occurred after rechallenge or dose reduction following initial improvement. Among the 55 patients, 7 were male (all olaparib-related), and 48 were female, yielding a male-to-female ratio of 1:6.8. Ages ranged from 36 to 82 years (mean 61.02 ± 10.76 years), and 47 patients (85.45%) were over 50 years of age, as presented in Figure 2.

**FIGURE 2:**
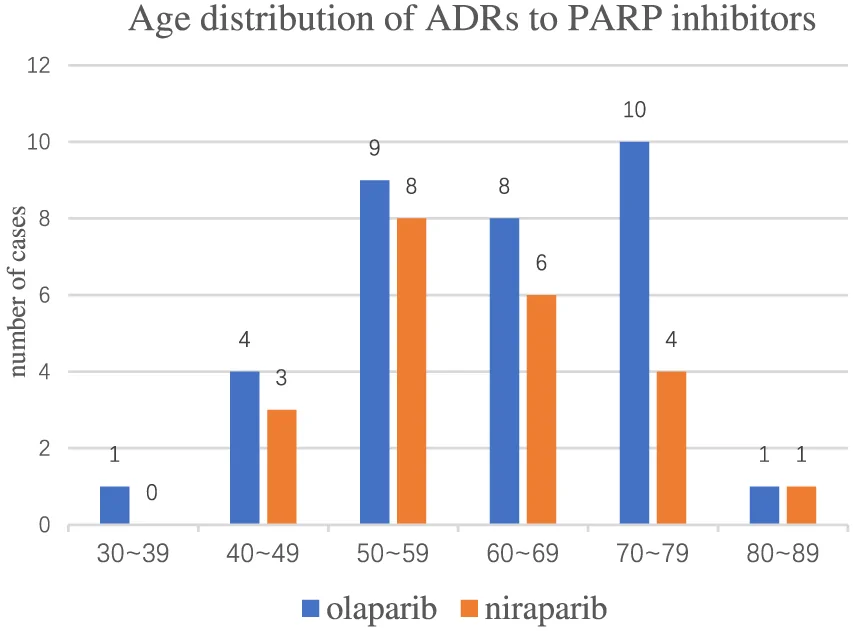
Age distribution of patients with ADRs induced by olaparib and niraparib.

### 3.4 Indications and dosage regimens

Among the 33 olaparib-related ADRs, the underlying malignancies comprised OC in 22 cases, prostate cancer in 5, breast cancer in 3, pancreatic cancer in 2, and endometrial cancer in 1. With respect to dosing, the standard recommended dose (300 mg twice daily (bid)) was administered to 23 patients; 10 patients received dose-reduced regimens (such as 150 mg bid, or 300 mg in the morning and 150 mg in the evening) following ADR occurrence; 2 patients were placed on a high dose (400 mg bid); 1 patient received a substantially reduced dose (40 mg once daily (qd)); and dosing information was not reported for the remaining 7 patients. For the 22 niraparib-associated cases, 18 were treated for OC, 2 for fallopian tube cancer, and 2 for breast cancer. Regarding dosage, 9 patients were given the standard recommended dose (300 mg qd); 5 patients underwent dose reduction to 200 mg or 100 mg qd after experiencing ADRs; 11 patients initiated therapy with an individualized dose of 200 mg qd; 1 patient received a low dose (100 mg qd); and no dose information was available for the remaining 1 patient. Detailed case characteristics are provided in Supplementary Table S1.

### 3.5 ADR occurrence time

Among the 68 ADR events documented across the 55 patients, the time from drug initiation to ADR occurrence ranged from 3 days (the shortest) to 21 months (the longest). For both olaparib and niraparib, the highest proportion of ADR events was observed within the first 30 days of treatment, accounting for 37.50% and 53.57% of the respective drug-related events. The occurrence time was not reported for 7 events. Detailed data are presented in Table 1.

**TABLE 1:** Time-to-onset distribution of ADRs.

Olaparib-related ADRs (40 events)			Niraparib-related ADRs (28 events)	
Time/d	Number of events (% of the proportion)	SOCs (number of examples)	Number of events (% of the proportion)	SOCs (number of examples)
1–30	15 (37.50)	Skin and subcutaneous tissue disorders (10) Blood and lymphatic system disorders (2) Respiratory, thoracic and mediastinal disorders (2) Hepatobiliary disorders (1)	15 (53.57)	Blood and lymphatic system disorders (10) Skin and subcutaneous tissue disorders (2) Musculoskeletal and connective tissue disorders (1) Respiratory, thoracic and mediastinal disorders (1) Nervous system disorders (1)
31–90	6 (15.00)	Respiratory, thoracic and mediastinal disorders (3) Blood and lymphatic system disorders (2) Skin and subcutaneous tissue disorders (1)	5 (17.86)	Respiratory, thoracic and mediastinal disorders (2) Blood and lymphatic system disorders (2) Nervous system disorders (1)
91–180	10 (25.00)	Respiratory, thoracic and mediastinal disorders (4) Hepatobiliary disorders (3) Immune system disorders (1) Blood and lymphatic system disorders (1) Neoplasms benign, malignant and unspecified (1)	1 (3.57)	Blood and lymphatic system disorders (1)
>180	7 (17.50)	Respiratory, thoracic and mediastinal disorders (2) Blood and lymphatic system disorders (3) Skin and subcutaneous tissue disorders (1) Neoplasms benign, malignant and unspecified (1)	2 (7.14)	Blood and lymphatic system disorders (2)
Not mentioned	2 (5.00)	Skin and subcutaneous tissue disorders (1) Blood and lymphatic system disorders (1)	5 (17.86)	Blood and lymphatic system disorders (5)

Some cases may involve multiple organ systems simultaneously, and a given patient could experience more than one ADR, event; accordingly, the aggregate count across system organ classes exceeds the total number of patients.

### 3.6 ADR involvement system and clinical manifestations

The 68 ADR events were distributed across eight system organ classes. It must be emphasized that the proportional distribution of ADRs across organ systems below reflects only the composition of published case reports included in this study. Given the inherent publication bias of case reports—which prioritize rare, severe, and atypical adverse reactions—these proportions do not represent the real-world incidence rate of each toxicity, nor can they be interpreted as common side effects of the drugs. Within the included case set, skin disorders and interstitial lung disease were the predominant manifestations of olaparib-associated ADRs, followed by hepatic injury and haematological toxicity. In contrast, ADRs attributed to niraparib were highly concentrated in the hematopoietic system, with thrombocytopenia identified as the core toxicity, comprising over 70% of the niraparib-related episodes. The detailed distribution is shown in Table 2.

**TABLE 2:** Distribution of ADRs involving SOCs and main clinical manifestations.

​	Olaparib-related ADRs (40 events)		Niraparib-related ADRs (28 events)	
SOCs	Main manifestations (number of examples)	Number of events (% of the proportion)	Main manifestations (number of examples)	Number of events (% of the proportion)
Skin and subcutaneous tissue disorders	Erythema nodosum (4), cutaneous vasculitis (2), rash (2), cutaneous arteritis (1), purpuric drug eruption (1), pseudoporphyria (1), hand-foot syndrome (1), cutaneous small-vessel vasculitis (1)	13 (32.50)	DRESS syndrome (1), sweet syndrome (1)	2 (7.14)
Respiratory, thoracic and mediastinal disorders	ILD/pneumonitis (10, including 3 cases of hypersensitivity pneumonitis and 1 case of eosinophilic pneumonia), pulmonary embolism (1)	11 (27.5)	ILD (1), pulmonary embolism (1), drug-induced pneumonitis (1)	3 (10.71)
Blood and lymphatic system disorders	Anemia (4, including hypoproliferative anemia and macrocytic anemia), MDS (2), thrombocytopenia (2, including 1 case of ITP), grade 4 myelosuppression (1)	9 (22.50)	Thrombocytopenia (15, including 1 case of pure megakaryocytic thrombocytopenic purpura and 1 case of severe refractory thrombocytopenia), anemia (4, including macrocytic anemia and PRCA, Pancytopenia (1)	20 (71.43)
Hepatobiliary disorders	DILI (4)	4 (10.00)	—	—
Neoplasms benign, malignant and unspecified	Meningeal metastasis from pancreatic cancer (1), cutaneous metastasis of pancreatic cancer (1)	2 (5.00)	—	—
Immune system disorders	Seronegative inflammatory arthritis (1)	1 (2.50)	—	—
Nervous system disorders	—	—	Progressive dysgeusia (ageusia) (2)	2 (7.14)
Musculoskeletal and connective tissue disorders	—	—	Elevated creatine kinase (with myalgia and swelling) (1)	1 (3.57)

Abbreviations: ILD, interstitial lung disease; MDS, myelodysplastic syndrome; ITP, immune thrombocytopenia; DILI, Drug-induced liver injury; PRCA, pure red cell aplasia; DRESS, Drug reaction with eosinophilia and systemic symptoms.

### 3.7 ADR treatment and outcome

For olaparib-treated patients, 5 achieved improvement after drug discontinuation alone, 2 after dose reduction, and 16 after discontinuation plus symptomatic treatment. One patient improved with symptomatic management without treatment interruption. Death occurred in seven patients: 3 died due to ADR (1 following discontinuation plus symptomatic care, 1 after dose reduction with continued maintenance therapy, and 1 who died despite symptomatic treatment without drug cessation) and 4 died due to disease progression. For the remaining 2 patients, the outcome was not reported. For the 22 patients on niraparib, 1 showed improvement after drug discontinuation, and 1 improved following dose reduction. A total of 15 patients recovered after discontinuation combined with symptomatic management, while 4 patients improved with symptomatic treatment added without stopping the drug. In 1 patient, no improvement was observed despite discontinuation and symptomatic care. No deaths were attributed to niraparib, and no cases were documented with unspecified outcomes.

### 3.8 ADR correlation evaluation

The causality assessment of ADRs induced by PARPis was based on the scores reported by the original authors using the Naranjo ADR Probability Scale; for those cases without such scores, the assessment was performed by the investigators using the same scale. The results demonstrated that, among the 55 patients, the causality was classified as “probable” in 42 cases, “definite” in 8 cases (All eight definite-causality cases underwent rechallenge and met the positive criterion, with recurrence of the adverse reaction observed in every case upon re-administration), and “possible” in 5 cases. The detailed evaluation results are shown in Supplementary Table S1.

## 4 Discussion

Olaparib and niraparib are oral PARPis that target PARP1 and PARP2 in homologous recombination-deficient (HRD) cells (including those with BRCA1/2 mutations), exerting antitumor effects *via* synthetic lethality (Guo et al., 2018; Maiorano et al., 2023). As key targeted agents, they are indicated for the treatment of ovarian, fallopian tube, and primary peritoneal cancers, among other malignancies (Pujade-Lauraine et al., 2017), and have been shown to prolong progression-free survival (PFS) and improve objective response rates (Moore et al., 2018; Zhao et al., 2023). From a pharmacological point of view, PARPis share a similar mode of action but are different in many characteristics. The defining characteristics of the two PARPis are summarized in Table 3 (Sandhu et al., 2022; Valabrega et al., 2021).

**TABLE 3:** Pharmacological properties of PARPis.

PARPis	Olaparib	Niraparib
Chemical structure	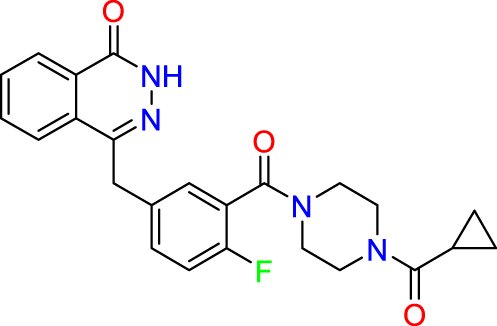	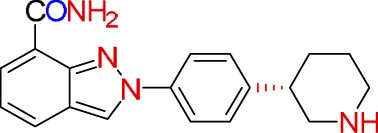
Dosage strengths	100 mg, 150 mg	100 mg, 200 mg, 300 mg
Dosage form	Tablet	Capsule
Storage	Up to 30 °C	Up to 25 °C
Starting dose	300 mg bid	200 mg qd; or 300 mg qd if the weight is 77 kg or the platelet is 150,000/µL
Volume of distribution (L)	158	1,074
Tmax (h)	1.5	3
Cmax (nM)	13,400	2,500
Half-life (h)	15	36
Plasma protein binding (%)	82	83
Clearance (L/h)	7	16.2
Metabolism	CYP3A4	Carboxylesterase
Bioavailability (%)	NA	73
Renal excretion (%)	44	47.5
On-target efficiency (median IC_50_ values in nM)	​	​
PARP-1	13	35
PARP-2	56	8
PARP-3	99	380
PARP-4	409	408
Off-target activity (median IC_50_ values in nM)	​	​
DYRK1A	—	297
DYRK1B	—	254
DAT	NA	51
NET	NA	239
SERT	NA	363
MATE1	5,500	—
MATE2-K	—	NA
5-HT4	NA	1,000
hERG	NA	1,000
Dose modification for ADRs	DL-1: 250 mg bid DL-2: 200 mg bid DL-3: Discontinue	If starting dose at 200 mg qd DL-1: 100 mg daily DL-2: Discontinue If starting dose at 300 mg qd DL-1: 200 mg qd DL-2: 100 mg qd DL-3: Discontinue
Drug-drug interactions	CYP3A4 inhibitors and inducers^a^	No clinically significant CYP-mediated interactions
Drug-food interactions	Grapefruit, star fruit, pomegranate, and seville orangese^b^	No clinically significant food effect

Abbreviations: “—”, no inhibition; NA, not available; bid, Twice daily; qd, Once daily; DL, dose level; PARP, Poly (ADP-ribose) polymerase; Cmax, Peak serum concentration; Tmax, Time taken to reach Cmax; IC_50_, Half maximal inhibitory concentration; DYRK, Dual specificity tyrosine phosphorylation-regulated kinase; DAT, dopamine transporter; NET, norepinephrine transporter; SERT, serotonin transporter; MATE1, Multidrug and toxin extrusion protein 1; MATE2-K, Multidrug and toxin extrusion protein 2 (kidney-specific); 5-HT4, 5-Hydroxytryptamine receptor 4; hERG, human Ether-à-go-go-Related Gene; CYP3A4, Cytochrome P450 3A4.

^a^ Strong CYP3A4 inhibitors and inducers are not recommended.

^b^ These fruits contain CYP3A4 inhibitors and are not recommended for concomitant use.

### 4.1 Relationship between ADRs and patient age and sex

Of the 68 ADR events observed in 55 patients, the majority occurred in those aged >50 years, accounting for 84.85% (olaparib) and 86.36% (niraparib), largely reflecting the demographic characteristics of the underlying malignancies. Furthermore, PARPis and their metabolites are primarily eliminated *via* renal and hepatic pathways, with approximately 44% of the dose excreted in urine and 42% in faeces (Rolfo et al., 2019). Given that hepatic and renal function often declines with age, and that tumor diseases are frequently accompanied by varying degrees of hepatorenal impairment, diminished gastrointestinal digestive capacity, and slowed metabolism, prolonged drug half-life and an increased proportion of ADR events may result. Due to the lack of detailed drug utilization data, we could not calculate incidence rates or definitively conclude that elderly patients have a higher ADR risk than younger patients. Nevertheless, in view of the relatively weaker hepatorenal function and the higher proportion of elderly patients in this cohort, it is recommended that clinicians closely monitor medication safety in this population.

Notable sex baseline imbalance existed between the two groups: the olaparib group included seven male prostate cancer patients and 26 females, while all niraparib cases were female, mainly driven by differences in approved indications. This imbalance is a non-negligible confounding factor for interpreting inter-group ADR differences. Sex-related physiological variations, concomitant medications and tumor backgrounds may jointly affect toxicity profiles; thus, the observed ADR differences cannot be directly attributed to sex-specific drug risks. Limited by the case report design and unbalanced baseline, this study cannot independently evaluate the effect of sex on PARP inhibitor-related ADRs. Age-wise, olaparib ADRs spanned 36–80 years, with the highest proportion (30.30%) in patients aged 70–79 years, suggesting that elderly patients may be more likely to experience severe pulmonary and haematological toxicities. Niraparib ADRs were more concentrated in the 50–69 age group (63.64%), with none reported in patients under 40 years, suggesting a younger onset age and lower representation in the very elderly. However, due to the small sample size, no definitive correlation between sex, age, and PARP inhibitor-associated ADRs could be established.

### 4.2 Relationship between ADR occurrence time and drug administration

The time to ADR onset in the 55 patients was found to be highly variable, with a wide span ranging from as short as 3 days to as long as 21 months after treatment initiation. Early-onset ADRs are a shared feature of both agents, and the first 1–30 days of administration were identified as a critical toxicity window, during which rapid-onset skin injury predominated in olaparib-treated patients, whereas haematological toxicity was more frequently noted in those receiving niraparib. In rechallenge settings, recurrent ADRs for both drugs were observed to occur at an early stage after therapy resumption (mostly within 1 week), suggesting that intensified safety monitoring should be performed during the initial period of re-administration.

Nevertheless, the temporal distribution patterns of the two drugs were markedly dissimilar. The ADRs associated with niraparib exhibited an early-concentrated unimodal distribution, with new-occurrence adverse events being significantly reduced after 91 days of treatment, indicating that the overall toxicity was characterised by rapid occurrence and early exposure, accompanied by a relatively lower risk of long-term organ damage. It was mentioned in a clinical trial of PARPis for OC that Most ADRs occur within the first 12 weeks of treatment (Friedlander et al., 2023), which is consistent with our findings. In contrast, a typical bimodal distribution was displayed by olaparib-related ADRs, with the first peak occurring at 1–30 days (accounting for 37.50%) and the second peak at 91–180 days (accounting for 25.00%), the latter being predominantly composed of delayed-occurrence organ toxicities such as ILD and DILI. Moreover, delayed haematological toxicity and pulmonary ADR were still reported beyond 180 days of treatment, reflecting a broader temporal span of ADRs and a more prominent risk of delayed organ damage. It should be clarified that the bimodal onset pattern of olaparib and the early unimodal distribution of niraparib are both derived from published case reports with complete time-to-onset data. These temporal characteristics are subject to follow-up duration bias and selective reporting bias: severe or clinically noteworthy early adverse events are more likely to be published, while mild or extremely delayed toxicities may be underreported. Thus, these patterns cannot be directly generalized to the full population of patients receiving PARP inhibitor therapy. Accordingly, it is suggested that haematological toxicity should be closely monitored during the early phase of niraparib therapy; for olaparib, however, cutaneous toxicity warrants initial surveillance, whereas hepatic and pulmonary toxicities warrant close monitoring during the later stages of olaparib therapy.

### 4.3 Relationship between ADRs and distribution of primary diseases

For both drug classes, ADRs were predominantly observed in ovarian cancer patients, accounting for 66.67% (22 cases) of olaparib-related ADRs and 81.82% (18 cases) of niraparib-related ADRs, which is in close alignment with the current clinical practice wherein PARPis are most widely applied for maintenance therapy of OC (Goldlust et al., 2024; Tuninetti et al., 2024). Additionally, 5 cases (15.15%) of olaparib-associated ADRs were reported in patients receiving postoperative maintenance therapy for prostate cancer. Although 2022 International Agency for Research on Cancer (IARC) global cancer statistics indicate that the global incidence of prostate cancer significantly exceeds that of OC (Bray et al., 2024), there is currently insufficient evidence to confirm that olaparib-treated prostate cancer patients outnumber those with OC, precluding a direct comparison of ADR incidence between indications. Therefore, it cannot be inferred at present that the proportion of ADR events associated with olaparib is higher in postoperative OC patients than in prostate cancer patients, and further investigation is warranted.

### 4.4 Relationship between ADRs and drug dosage

Among the 55 patients enrolled in this study, the recommended standard doses were most commonly used for both olaparib (300 mg bid in 23 cases, 69.70%) and niraparib (200 mg qd in 11 cases, 50.00%; 300 mg qd in 9 cases, 40.91%). Most ADR cases occurred in patients receiving standard therapeutic doses, which is likely attributable to the larger proportion of patients treated with these doses in clinical practice. Olaparib at 300 mg bid, which was most widely applied in clinical practice and provided sufficient long-term exposure, could induce ADRs involving multiple systems, including the skin, lungs, blood, and hepatobiliary tract. At a higher dose (400 mg bid), the risks of organ toxicities, such as hepatic and pulmonary toxicity, were further elevated. For niraparib, the highest haematological toxicity risk was observed at 300 mg qd, with a greater propensity for severe thrombocytopenia; the 200 mg qd dose, as a mainstream starting dose, was extensively used in clinical practice and accounted for the largest proportion of total ADR cases, affecting the haematological, respiratory, and nervous systems. A clear dose-response relationship for ADRs could not be established from our data. Nevertheless, it could be inferred that dose reduction, which lowers plasma concentrations, might decrease potential adverse reactions without compromising therapeutic efficacy. In a study of 87 *BRCA*-mutated OC patients treated with olaparib, dose reduction (from 600 mg/day to 400 mg/day) was performed in 52% of patients. The median PFS after dose reduction was 32 months, which was superior to the 27 months observed in the non-reduced group (Boccia et al., 2026). Another study indicated that a high dose of olaparib (400 mg bid) did not yield better outcomes for OC, with an overall tumor response rate of only 26.2%, and the vast majority of patients experienced ADRs (Kaufman et al., 2014). Regarding niraparib, the NEWTON trial (Phase II) compared the standard dose (300 mg qd) with an individualised starting dose (200 mg qd). The incidence of grade ≥3 thrombocytopenia was significantly reduced from 41.7% in the standard-dose group to 4.2% in the individualised-dose group, without affecting PFS (Colombo et al., 2026). These results suggested that dose reduction was a feasible option for patients experiencing treatment-related adverse events. In our case series, 4 olaparib-treated patients and 1 niraparib-treated patient experienced no ADR recurrence following dose reduction. Furthermore, for niraparib-associated thrombocytopenia, the co-administration of avatrombopag could be added to prevent the progression of related ADRs. Clinicians are therefore advised to pay close attention to the tolerability of patients at higher risk of elevated plasma concentrations. For patients who develop ADRs after standard-dose treatment, if disease progression does not necessitate complete drug discontinuation, dose reduction of the PARP inhibitor and continued observation may be considered.

### 4.5 Mechanisms of ADR generation and clinical manifestations

#### 4.5.1 ADR of blood and lymphatic system

Among the 68 ADR events, 29 (42.65%) involved haematological system disorders, comprising 9 olaparib events and 20 niraparib events. Haematological toxicity was the predominant ADR type for niraparib, with a significantly higher proportion than for olaparib. This finding is consistent with the conclusions derived from the analysis of the FAERS database (Tian et al., 2022). In terms of clinical manifestations, the haematological toxicity profiles of the two drugs were clearly differentiated. For olaparib, anaemia was the most frequently observed haematological toxicity, including subtypes such as macrocytic anaemia, hypoproliferative anemia, and MDS, with erythroid haematopoietic impairment and long-term bone marrow clonal abnormalities being the prominent characteristics overall. MDS is regarded as the most serious long-term adverse event of PARPis, and a significantly elevated risk of MDS was clearly demonstrated in a meta-analysis of 5,693 patients treated with these agents (Morice et al., 2021). For niraparib, thrombocytopenia was the core manifestation, with 15 events observed, encompassing severe thrombocytopenia, pure megakaryocytic thrombocytopenic purpura, immune thrombocytopenia, and other subtypes, followed by anaemia; megakaryocytic haematopoietic impairment was considered its most hallmark toxicity feature. A meta-analysis of 29 randomised controlled trials involving over 9,000 patients confirmed that PARPis significantly increased the risks of all-grade anaemia, neutropenia, and thrombocytopenia (Wang and Li, 2021). The common subtypes of PARP include PARP1, PARP2, and PARP3. In addition to its role in DNA repair, PARP1 was shown to be involved in the regulation of erythroid differentiation in the haematopoietic system, playing an important role in the regulation of erythropoiesis (Hopkins et al., 2019; Madariaga et al., 2020). Furthermore, PARP2 was also found to be expressed in the megakaryocytic lineage, and platelets are derived from the cytoplasmic fragmentation of mature bone marrow megakaryocytes; thus, PARP was considered to exert a regulatory effect on haematopoietic stem cells (de Botton et al., 2002; Farrés et al., 2015). By inhibiting PARP1 and PARP2, PARPis reduce platelet formation and erythroid differentiation, which are the possible mechanisms underlying thrombocytopenia and anaemia. Among them, the myelosuppressive effect of niraparib was stronger than that of olaparib, especially with a significantly elevated risk of thrombocytopenia, which was regarded as the core reason for the difference in haematological toxicity proportions between the two drugs (Luo et al., 2022). In addition, the difference in platelet toxicity might also be related to the volume of distribution, with niraparib having a volume of distribution of 1074 L, compared with only 158 L for olaparib (Table 3). Therefore, the platelet toxicity of niraparib was significantly higher (Valabrega et al., 2021).

At the therapeutic level, stratified and precise interventions should be implemented according to the type, grade, and underlying mechanisms of toxicity. For mild-to-moderate thrombocytopenia, dose reduction should be undertaken initially, along with close haematological monitoring. For niraparib-induced thrombocytopenia, early combination therapy with thrombopoietin receptor agonists (TPO-RAs), such as avatrombopag, may be administered, so that platelet levels can be raised while the drug dose is maintained as much as possible, thereby ensuring the continuity of anticancer treatment. The efficacy and safety of this combination strategy have already been demonstrated in relevant case series (Gabrail and Smith, 2020). Furthermore, the protocols for drug rechallenge and switching should be standardised by clinicians. The present case series suggests that, after haematological toxicity has resolved, strict dose-reduction principles should be followed upon re-administration. For niraparib, a gradual dose escalation may be attempted under the cover of TPO-RA supportive therapy; for olaparib, however, rechallenge should be prohibited if severe myelosuppression or suspected MDS has occurred. In patients who cannot tolerate the haematological toxicity of a single PARP inhibitor, a switch to the other agent may be attempted, leveraging the differentiated toxicity profiles of the two drugs. However, drug switching requires full consideration of anti-tumour efficacy and patient tolerability. Given limited case-based evidence, such switching should not be regarded as a routine clinical recommendation.

#### 4.5.2 ADR of skin and subcutaneous tissue

Skin and subcutaneous tissue disorders were involved in a total of 15 events, of which 13 were attributed to olaparib (13/40, 32.50%), representing the highest proportion among all ADR types for this drug, and being far higher than the 2 events observed for niraparib (2/28, 7.14%). This indicates that the risk of cutaneous toxicity is more prominent for olaparib. In terms of clinical manifestations, the cutaneous toxicity profiles of the two drugs were clearly differentiated. Olaparib-associated skin ADRs were characterized by a wide variety of subtypes, including cutaneous small-vessel vasculitis, erythema nodosum, purpuric drug eruption, pseudoporphyria, hand-foot syndrome, and non-specific rashes. In contrast, although the incidence of niraparib-related cutaneous adverse events was lower, their severity was significantly higher. Both of the 2 reported cases were classified as severe cutaneous adverse reactions (SCAR), namely DRESS syndrome and Sweet syndrome. Such adverse events, which may involve multiple organ systems and even become life-threatening, are considered rare but serious toxicities among PARPis. The mechanism underlying PARP inhibitor-induced cutaneous toxic reactions remains unclear. It is currently believed to be mainly associated with immune-mediated inflammatory responses (Stephan and Magro, 2025). It has been suggested in some studies that PARPis may alter the immune balance, shifting inflammatory responses from a Th2-dominant to a Th1-dominant phenotype. Such a shift might promote the development of cutaneous adverse reactions such as vasculitis (Mustapha et al., 2006; Srivastava et al., 2005). In addition, the molecular structures of some PARPis may confer photosensitising properties. Although related investigations have focused primarily on rucaparib, they indicate potential risks within this drug class. The molecular structure of rucaparib enables it to absorb UVA radiation, thus exhibiting potential phototoxicity, which may cause damage to proteins and DNA in the skin (Mateos-Pujante et al., 2022). This provides a reference for understanding the photosensitivity reactions associated with other PARPis, including olaparib and niraparib. Clinicians are therefore advised to recommend, when necessary, the use of sunscreen, hats, and protective clothing for sun protection during treatment with these agents, so that phototoxic skin reactions can be avoided.

#### 4.5.3 Respiratory, thoracic and mediastinal ADR

In the 55 patients enrolled in this study, respiratory system disorders were observed, including 11 events of ILD and 2 events of pulmonary embolism, among which 1 patient died 5 months later without treatment discontinuation. In a previous randomised double-blind study of olaparib for metastatic prostate cancer, an incidence of ILD of 2% was reported (Roubaud et al., 2022). In the FDA Adverse Event Reporting System (FAERS) database, the incidence of PARP inhibitor-associated pneumonitis was reported to be 0.97%. However, among the 79 patients with pneumonitis, 13 deaths (16%) were recorded (Ma et al., 2021). Therefore, when PARPis are administered, the associated pneumonitis warrants significant clinical attention because of its high mortality rate. At present, the mechanism underlying PARP inhibitor-induced respiratory toxicity remains unclear; it has been suggested by some studies that this might be mediated through altered sensitivity to oxidative stress affecting pulmonary inflammation, since PARP has been implicated in airway hyperresponsiveness and airway remodeling (Zhang et al., 2021).

#### 4.5.4 Hepatobiliary ADR

In the present study, hepatobiliary disorders were involved in a total of 4 events (5.88%), and all were observed in the olaparib group. The hepatobiliary ADRs all featured drug-induced liver injury as the core manifestation, with markedly elevated serum alanine aminotransferase and aspartate aminotransferase levels being the main laboratory characteristics. In terms of the temporal pattern of onset, olaparib-induced liver injury occurred mostly at 3.5–4 months after treatment initiation, and was therefore classified as a delayed-type ADR, which was closely associated with the accumulation of metabolites after long-term drug exposure and the gradual activation of immune-inflammatory responses. Rucaparib and olaparib inhibit MATE1 (and MATE2 for rucaparib), reducing renal creatinine secretion and potentially elevating serum creatinine (Liao et al., 2020). Additionally, olaparib is primarily metabolized by CYP3A4 with multiple reactive metabolites, and long-term accumulation may trigger metabolic idiosyncratic liver injury. In contrast, niraparib does not inhibit MATE1 (Table 3) and is mainly metabolized by carboxylesterases with fewer hepatotoxic intermediates, which may partly explain the inter-drug difference in hepatorenal toxicity (McCormick and Swaisland, 2017). Clinicians are therefore advised to be vigilant for delayed liver injury ADRs when olaparib is used, whereas niraparib may be preferred as the preferred agent for patients with hepatic insufficiency.

#### 4.5.5 Neoplasms benign, malignant and unspecified and immune system ADRs

The only immune system ADR induced by olaparib was seronegative inflammatory arthritis, which manifested as unilateral wrist and ankle joint swelling and pain with limited movement and morning stiffness, occurring 6 months after drug initiation. PARP inhibition was previously considered an effective therapeutic approach for some inflammatory rheumatic diseases. It has been shown in animal studies that PARPis can suppress fibroblast proliferation and alleviate synovitis in collagen-induced arthritis mouse models (Miesel et al., 1995), which may be primarily attributed to the reduction of transcription factors (nuclear factor-κB, activator protein-1) and the activation of signalling pathways, leading to downregulation of pro-inflammatory mediator expression (Mabley et al., 2001). This contrasts with our case findings and presents a paradox. The underlying mechanism of olaparib-induced arthritis therefore remains unclear and may be attributed to immune system dysregulation, triggering of the proinflammatory cytokine cascade, and B-cell reactivation (Fusun and Senol, 2019).

Two cases of tumor metastasis associated with olaparib were both observed in patients with BRCA-mutated pancreatic cancer receiving maintenance therapy, presenting as leptomeningeal metastasis and cutaneous metastasis, respectively, and occurring during the maintenance phase at 4–12 months after treatment initiation. The etiology of these events remains controversial, and whether they are attributable to natural disease progression or to drug-induced alterations in the invasive phenotype of the tumor cannot be clearly determined; further validation by large-scale cohort studies is still required. In contrast to olaparib, no case reports of niraparib monotherapy-induced autoimmune joint injury or tumor metastasis to unusual sites have been reported to date, suggesting that potential inter-class differences between the two drugs are implicated with respect to immunomodulatory effects and impact on tumor biological behavior. Based on the above findings, attention should be paid to newly developed immune-related symptoms, such as joint swelling and pain, in patients treated with olaparib; inflammatory markers and imaging evaluations should be promptly performed for early intervention. Meanwhile, for patients on long-term maintenance therapy for rare tumor types including pancreatic cancer, the possibility of metastasis to unusual sites should be kept in mind.

#### 4.5.6 Nervous system and musculoskeletal and connective tissue ADRs

In the present study, progressive dysgeusia was identified as the core manifestation of nervous system ADRs, with bitter taste being perceived at approximately 90 days after treatment initiation and gradually progressing to complete taste loss, which was spontaneously resolved after drug discontinuation. It was revealed in an analysis based on the FAERS database that neurotoxic reactions might be more frequently observed in patients treated with niraparib than in those receiving olaparib (Tian et al., 2022). Niraparib is a relatively potent dopamine transporter (DAT) inhibitor, with an IC_50_ value of 51 nM (Table 3). Moreover, DAT, as a dopamine transporter protein, has been demonstrated to play a significant role in schizophrenia and delirium, and thus has been considered to account for the commonly observed neurological disorders associated with niraparib (Hahn and Blakely, 2002). In addition, a higher risk of neurotoxicity was attributed to niraparib due to its greater blood–brain barrier penetration (Friedlander et al., 2023). These findings are consistent with the absence of olaparib-related neurotoxicity observed in our study.

As for musculoskeletal ADRs, acute onset of bilateral upper extremity soreness and swelling, accompanied by facial edema and markedly elevated creatine kinase levels, were observed, occurring as early as 6 days after drug administration, which was classified as early-occurrence drug-related muscle injury. It has been reported that the overall incidence of myalgia was 11.3% and that of arthralgia was 14.9% for olaparib; for niraparib, the overall incidence of myalgia was 13.4% in the fixed starting-dose group (300 mg qd), whereas it was only 7.9% in the individualised starting-dose group (200 or 300 mg qd) (Bao et al., 2021; Mirza et al., 2020), which remains unexplained.

## 5 Limitations

This study has several limitations. First, only published case reports were included, which tend to prioritize severe or atypical ADRs, leading to inherent publication bias. Second, the small sample size and variable reporting quality across studies precluded calculation of true ADR incidence and may have introduced statistical bias. Finally, the lack of population-level drug utilization data limits the generalizability of our findings. In addition, some of the cases included in this study may have had incomplete information due to restrictions on article length, so that the proportion of ADR events could not be fully determined. The level of detail in the description of patient information varied across case reports; hence, the statistical analysis results might be biased to some extent, and the accuracy of the causality assessment might also be affected. These mechanistic interpretations for rare ADRs are built on biologically sound reasoning, yet definitive mechanistic validation cannot be achieved given the limited number of individual case reports. Further clarification of the toxicity differences and underlying mechanisms between the two classes of PARPis is still warranted through large-sample, multicentre real-world studies and basic experimental research.

## 6 Conclusion

PARPis are widely used in maintenance therapy for breast, ovarian, and prostate cancers, but ADRs increase with the expansion of clinical application. Based on a systematic analysis of published case reports, the characteristics, distributions, and mechanisms of ADRs for olaparib and niraparib were summarized. Common toxicities were identified in hematologic, cutaneous, and respiratory systems. However, significant heterogeneity was observed: olaparib showed higher proportions of cutaneous, respiratory, and hepatobiliary ADRs, including delayed myelodysplastic syndrome; niraparib toxicities were predominantly hematologic (thrombocytopenia), with neuromuscular effects (dysgeusia, CK elevation). Therefore, individualized drug selection and monitoring strategies should be guided by patient status and tolerance. These findings provide evidence-based references for safe use and refined ADR management, reducing toxicity while preserving antitumor efficacy, ultimately improving patient adherence and quality of life.

## Data Availability

The original contributions presented in the study are included in the article/Supplementary Material, further inquiries can be directed to the corresponding author.
