# Supplementary material for: A retrospective analysis of adverse drug reaction case reports for PARP inhibitors: a comparative study of olaparib and niraparib
Source: Front Pharmacol. 2026 Sep 24;17:1935594. doi: 10.3389/fphar.2026.1935594 (PMC13646078; doi:10.3389/fphar.2026.1935594)
Supplement: Supplementary file 1 [file Table1.docx]

**Table 1 Characteristics of included patients**

| Literature source | Patient  Sex, Country | age | Primary disease | ADR Diagnosis | Clinical manifestations | Dosage when ADR occurs | Time to ADR onset | Intervention measures | Outcome | Recovery time | Drug rechallenge | Combined medication | Causality assessment |
| --- | --- | --- | --- | --- | --- | --- | --- | --- | --- | --- | --- | --- | --- |
| **Olaparib** | | | | | | | | | | | | | |
| 1 Ishii et al^1^ | Male  Japan | 70 | PrCa | Pulmonary embolism. | dyspnea, chest pain | 300 mg bid | 7 months | Discontinue medication, Apixaban 5mg bid, oxygen therapy | Death (due to disease progression) | 35 days | Yes, Olaparib 150 mg bid again after 7 months did not result in pulmonary embolism | None | Probable |
| 2.1 Ishimoto et al^2^ | Female  Japan | 72 | OC | ILD (Hypersensitivity pneumonitis) | Fever and fatigue | 300 mg bid | 4 months | Discontinue medication, treat with glucocorticoids | Improved | 1 month | No | bevacizumab | Possible |
| 2.2 | Female  Japan | 51 | OC | ILD (Hypersensitivity pneumonitis) | Continuous fever and fatigue | 300 mg bid | 9 months | Discontinue medication | Improved | 1 month | No | Flunitrazepam | Probable |
| 2.3 | Female  Japan | 78 | OC | ILD (Hypersensitivity pneumonitis) | Fever, cough, and dyspnea | 300 mg bid | 14 days | Discontinue medication，oxygen therapy, injection of methylprednisolone+piperacillin tazobactam+azithromycin | Improved | 2 months | No | None | Probable |
| 3 Sun et al^3^ | Male  China | 61 | PrCa | ILD (Eosinophilic pneumonia) | low-grade fever, cough, chest tightness, shortness of breath | 300 mg bid | 5 months | Discontinue medication，Methylprednisolone 40 mg intravenous infusion, once a day, meropenem for anti-infection | Improved | 14 days | No | None | Probable |
| 4 Kaitsumaru et al^4^ | Male  Japan | 67 | PrCa | ILD | High fever, dyspnea | 250 mg bid | 2 months | Corticosteroid pulse therapy | Death (due to disease progression) | - | No | - | Probable |
| 5.1 Hattori et al^5^ | Female  Japan | 52 | OC | ILD | High fever, dyspnea | 300 mg bid | 125 days | Discontinue medication and take oral prednisone | Improved | 70 days | No | - | Probable |
| 5.2 | Female  Japan | 76 | OC | ILD | Intermittent low-grade fever, dyspnea | 300 mg bid | 65 days | Stop medication and take oral prednisone+piperacillin tazobactam for anti-infection | Death (due to ADR) | - | No | - | Probable |
| 5.3 | Female  Japan | 73 | OC | ILD | High fever, fatigue, respiratory failure | 300 mg bid | 55 days | Stop medication and take oral prednisone+piperacillin tazobactam for anti-infection | Improved | - | No | - | Probable |
| 6 Si et al^6^ | Male  China | 61 | PrCa | ILD | High fever, cough, dyspnea | 300 mg bid | 106 days | Stop taking the medication and take methylprednisolone orally | Improved | 22 days | Yes, Olaparib 300 mg bid again, high fever (ILD) occurrence after 2 days. After 37 days, Olaparib was at 300 mg in the morning and 150 mg in the night, no ADR occurrence | - | Definite |
| 7 Tufoni et al^7^ | Female  Italy | 61 | OC | DILI | ALT and AST levels significantly increased | 400 mg bid | 4 months | Two weeks of discontinuation without improvement, oral prednisone | Improved | - | No | - | Probable |
| 8 Zhu et al^8^ | Female  Canada | 70 | OC | DILI | ALT and AST levels significantly increased | 300 mg bid | 105 days | Discontinue medication | Improved | 20 days | Yes, Olaparib 150 mg bid again, severe Grade 4 liver damage occurred 12 days later. withdrawn and switch to Niraparib 100 mg bid without any ADRs | None | Definite |
| 9 Alshelleh et al^9^ | Female  United States | 56 | EC | DILI | ALT and AST levels significantly increased, with jaundice and mild tenderness in the upper right abdomen | - | 3.5 months | Discontinue medication and take oral prednisone | Improved | 28 days | No | None | Probable |
| 10 Kobak^10^ | Female  Turkey | 60 | OC | Seronegative inflammatory arthritis | Left wrist and ankle joint pain, swelling, limited mobility with morning stiffness Significant increase in ESR and CRP | - | 6 months | Without stopping the medication + Low dose glucocorticoids+oral hydroxychloroquine | Improved | 3 months | - | None | Probable |
| 11 Álvarez-Salafranca et al^11^ | Female  Spain | 58 | BC | Cutaneous vasculitis | Non-ulcerative nodules with multiple erythematous rashes and mild pain in the limbs (forearm, lower limb) | 150 mg bid | 7 days | Without stopping the medication，Skin symptoms persist. After stopping the medication one year later, the condition improved | Improved | - | No | No | Probable |
| 12 Wheelden et al^12^ | Female  United States | 45 | BC | Erythema nodosum | Multiple erythematous pain nodules in both lower limbs, accompanied by lower limb edema | 300 mg bid | 3 days | Stop taking medication and administer nonsteroidal anti-inflammatory drugs | Improved | 7 days | Yes， After attempting 250 mg bid and 200 mg bid in sequence, nodular erythema appeared after 2 days | None | Definite |
| 13 Clark et al^13^ | Female  Philippines | 71 | OC | Pseudoporphyria | Pruritic rash, with the skin lesions evolving from erythema to blisters to hard papules | - | - | Discontinue medication, take oral hydroxyzine to relieve itching, and apply topical corticosteroids | Improved | - | No | None | Probable |
| 14 Saito et al^14^ | Female  Japan | 69 | BC | Erythema nodosum | Multiple tender erythematous infiltrative plaques on the extensor side of both calves, accompanied by difficulty walking | 300 mg bid | 4 days | Discontinue medication and take oral prednisone | Improved | 7 days | No，the regimen was subsequently switched to S-1 (tegafur/gimeracil/oteracil). | None | Probable |
| 15 Iinuma et al^15^ | Female  Japan | 53 | OC | Cutaneous arteritis | Multiple slightly raised erythema with unclear boundaries and a diameter of about 1cm on the back of the forearm and front of the thigh | 300 mg bid | 4 days | Discontinue medication | Improved | 7 days | No | None | Probable |
| 16 Costa et al^16^ | Female  Brazil | 48 | OC | Hand-foot syndrome | Red spots appear on the palms and soles of the feet, accompanied by dry and flaky skin, as well as local pain, irritation, and discomfort | - | 3 months | Without stopping the medication，External moisturizing agents, medium-potency topical corticosteroids | Death (due to ADR) | - | No | None | Probable |
| 17 Fukui et al^17^ | Female  Japan | 80 | OC | Cutaneous vasculitis | Multiple purpura in the left lower limb, with swelling in both lower limbs and multiple purpura accompanied by subcutaneous induration after progression | 40 mg qd | 1 month | Discontinue medication | Improved | 7 days | No | - | Probable |
| 18 Sekine et al^18^ | Male  Japan | 70 | PrCa | Purpuric drug eruption | Painless, non-raised scattered light purple bruising on fingers and fingertips | 300 mg bid | 1 month | Not discontinued, reduced to 150 mg bid, topical moisturizer and vitamin E ointment | Improved | - | No | None | Probable |
| 19 Gou et al^19^ | Female  Japan | 70 | OC | Rash complicated with anemia | Left back rash with swelling; Anemia, neutropenia | 300 mg bid | 11 months | Discontinue medication and administer topical corticosteroids and oral antihistamines | Improved | 14 days | Yes, Olaparib again, a rash immediately appeared, and it improved after stopping the medication again | None | Definite |
| 20 Stephan et al^20^ | Female  United States | 36 | OC | Cutaneous small-vessel vasculitis | Red patches on the anterior medial side of both calves, surface telangiectasia of skin lesions, and edema in the left lower limb | 300 mg bid | 1 month | Medication continued, only topical corticosteroid ointment for local symptomatic treatment | - | - | - | None | Probable |
| 21 Wang et al^21^ | Female  China | 51 | OC | Grade 4 bone marrow suppression | Grade 4 severe anemia, Grade 4 neutropenia, Grade 4 thrombocytopenia | 300 mg bid | 140 days | Discontinue medication, recombinant human erythropoietin+recombinant human thrombopoietin+recombinant human granulocyte colony-stimulating factor | Improved | 16 days | Yes, Olaparib was administered at a morning dose of 300 mg and a night dose of 150 mg without any ADRs | None | Probable |
| 22 Wang et al^22^ | Female  China | 55 | OC | Thrombocytopenia | Platelet count drops to a minimum of 57 × 10^9^/L | 300 mg bid | 45 days | Stop taking the medication and take Eltrombopag orally | Improved | - | Yes，Olaparib 150 mg bid, ADRs occurrence again | None | Definite |
| 23 Yao et al^23^ | Female  China | 58 | OC | MDS | Progressive pancytopenia, continuous decrease in white blood cells, hemoglobin, and platelets, with the lowest platelet count dropping to 2 × 10^9^/L | 300 mg bid | 25 days | Reduce to 300 mg in the morning and 150 mg in the evening and continue using for 12 months | Death (due to ADR) | - | - | None | Probable |
| 24 Yuan et al^24^ | Female  China | 69 | OC | ITP | Progressive decrease in platelet count | 300 mg bid | 14 days | Stop medication, platelet transfusion | - | - | No | None | Probable |
| 25 Linn et al^25^ | Female  China | 42 | OC | MDS | Severe pancytopenia, severe anemia, severe thrombocytopenia | - | 21 months | Infusing red blood cells and platelets, combined with hematopoietic growth factors for symptomatic support, to correct severe blood cell reduction | Improved | 4 months | No | Paclitaxel, carboplatin | Probable |
| 26 Dereme et al^26^ | Female  Switzerland | 71 | OC | Hyporegenerative anemia | Progressive anemia with a minimum hemoglobin level of 38 g/L (CTCAE Grade 4), accompanied by severe reduction in reticulocytes | 300 mg bid | 7 months | Stop medication and administer red blood cell transfusion | Improved | 3 months | Yes，the dosage was reduced to 150 mg qd, but anemia reappeared (time not mentioned) and the medication was permanently discontinued | Paclitaxel, Bevacizumab | Probable |
| 27 Shammo et al^27^ | Female  United States | 61 | OC | Macrocytic anemia | Severe folate deficiency, with hemoglobin levels dropping to a minimum of 6.6 g/dL | 300 mg bid | 35 days | Not discontinued, reduce to 200 mg bid, then reduce to 100 mg bid and supplement with folic acid | Improved | - | - | None | Probable |
| 28.1 Assaf et al^28^ | Male  Belgium | 48 | PaCa | Meningeal metastasis | - | - | 12 months | symptomatic treatment | Death (due to disease progression) | - | - | None | Possible |
| 28.2 | Male  Belgium | 54 | PaCa | Cutaneous metastasis | - | - | 4 months | symptomatic treatment | Death (due to disease progression) | - | - | None | Possible |
| **Niraparib** | | | | | | | | | | | | | |
| 29 Shono et al^29^ | Female  Japan | 73 | FTC | ILD | Cough, dyspnea after physical activity, rales in the lower right lung | 200 mg qd | 47 days | Discontinue medication, use levofloxacin for anti-infection and glucocorticoid therapy | Improved | 192 days | No | Vilanterol, Fluticasone nasal spray | Probable |
| 30 Wei et al^30^ | Female  China | 55 | OC | Pulmonary embolism | Dyspnea, chest pain, lower abdominal pain | 200 mg qd | 75 days | Discontinue medication and undergo anticoagulant therapy | Improved | 7 days | - | None | Probable |
| 31 McLaren et al^31^ | Female  United Kingdom | 58 | OC | Thrombocytopenia | Thrombocytopenia | 200 mg qd | 21 days | Discontinue medication | Improved | 21 days | Yes, Niraparib 100 mg qd again. Drug induced pneumonia appeared 7 days later, and the medication was permanently discontinued. | - | Probable |
| 32 Zhao et al^32^ | Female  China | 67 | OC，FTC with peritoneal carcinomatosis | Creatine kinase elevation | Swelling and soreness in both upper limbs, mild soreness in both lower limbs, facial swelling, intermittent palpitations, and general fatigue | 300 mg qd | 6 days | Stop taking the medication and receive symptomatic treatment with celecoxib and spironolactone | Improved | 12 days | No | Felodipine tablets, Irbesartan tablets | Probable |
| 33 Vázquez-Barrera et al^33^ | Female  Spain | 73 | OC with peritoneal carcinomatosis | DRESS syndrome | Diffuse confluent papules, severe pruritus, facial edema | 200 mg qd | 20 days | Stop taking medication and treat with glucocorticoids | Improved | 12 days | No | Letrozole, calcitriol, apixaban | Probable |
| 34 Badiner et al^34^ | Female  United States | 57 | OC | Sweet syndrome | Widespread erythema, blisters, and papules throughout the body, accompanied by pain and itching, affecting the trunk, limbs, neck, and back | - | 4 days | Stop medication and receive symptomatic treatment with glucocorticoids | Improved | 7 days | No | - | Probable |
| 35 Lei et al^35^ | Female  China | 53 | OC | Dysgeusia | Progressive taste disorder, initially characterized by bitter taste, progresses to complete loss of taste in the later stages | 200 mg qd | 90 days | Not stopped taking medication, given methylcobalamin and vitamin B12 to neurotrophic support | Not improved | - | After taking the medication continuously for 9 months, severe anemia appeared. withdrawn and receiving blood transfusion, the anemia was corrected and taste was restored. After 5 months, Niraparib 200 mg qd was taken again, and taste loss occurred again after 1 month | Insulin degludec/insulin aspart and Linagliptin | Probable |
| 36.1 Gabrail et al^36^ | Female  United States | 43 | BC | Thrombocytopenia | Thrombocytopenia | 300 mg qd | 30 days | Untreated, significant increase in platelet count was observed after combined use of avatrombopag | Improved | 21 days | - | None | Probable |
| 36.2 | Female  United States | 44 | BC | Thrombocytopenia | Thrombocytopenia | 300 mg qd | 21 days | Stop taking the medication and treat with avatrombopag | Improved | 7 days | Yes, Subsequently, the combination therapy of Niraparib 300 mg qd and avatrombopag was resumed without any ADRs | None | Definite |
| 36.3 | Female  United States | 61 | OC | Thrombocytopenia | Thrombocytopenia | 300 mg qd | - | Not discontinued, reduce dosage to 200 mg qd and add avatrombopag | Improved | - | Yes, Niraparib 300 mg qd again, there was a recurrence of thrombocytopenia. However, when combined with avatrombopag, no ADR recurred | None | Definite |
| 36.4 | Female  United States | 63 | OC，  FTC with peritoneal carcinomatosis | Thrombocytopenia | Thrombocytopenia | 300 mg qd | - | Not stopped taking medication, treated with avatrombopag | Improved | - | - | bevacizumab | Probable |
| 36.5 | Female  United States | 52 | OC | Thrombocytopenia | Thrombocytopenia | 200 mg qd | - | Not stopped taking medication, treated with avatrombopag | Improved | - | - | None | Probable |
| 36.6 | Female  United States | 75 | OC | Thrombocytopenia | Thrombocytopenia | 300 mg qd | - | Not stopped taking medication, treated with avatrombopag | Improved | - | - | None | Probable |
| 37 Jin et al^37^ | Female  China | 43 | OC | Thrombocytopenia | Thrombocytopenia | 300 mg qd | 25 days | Discontinue medication, subcutaneous injection of recombinant human thrombopoietin and oral avatrombopag | Improved | 11 days | Later switching to olaparib did not result in thrombocytopenia | - | Probable |
| 38 Chen et al^38^ | Female  China | 75 | OC | Thrombocytopenia | Thrombocytopenia | 200 mg qd | 18 days | Stop medication, recombinant human thrombopoietin | Improved | 11 days | No | Alendronate Sodium Vitamin D3 Tablets | Probable |
| 39 Guo et al^39^ | Female  China | 63 | OC with peritoneal carcinomatosis | AATP | Thrombocytopenia accompanied by nosebleeds and subcutaneous bleeding in both lower limbs | 200 mg qd | 21 days | Stop taking the medication and use recombinant human thrombopoietin, glucocorticoids, Hetrombopag, and avatrombopag successively to increase platelets | Not improved | - | No | None | Probable |
| 40 Xu et al^40^ | Female  China | 57 | OC，FTC | Thrombocytopenia | Thrombocytopenia, neutropenia, and leukopenia | 100 mg qd | 19 days | Discontinue medication and subcutaneous injection of multiple hematopoietic growth factors (leukocyte-stimulating, thrombopoietic and erythropoietic agents) | Improved | 18 days | No | - | Probable |
| 41 Kobayashi et al^41^ | Female  Japan | 57 | OC | Macrocytic anemia | Red blood cells, white blood cells, neutrophils, and platelets all decrease | 200 mg qd | 40 days | Stop medication and administer platelet transfusion | Improved | 14 days | Yes, Niraparib 100 mg qd again, no ADRs occurrence. But increase the dose to 200 mg qd, Grade 3 anemia reappeared | None | Definite |
| 42 Yücel et al^42^ | Female  Turkey | 65 | FTC | PRCA | Hemoglobin reduction, severe decrease in reticulocytes, and almost complete loss of bone marrow erythroid precursor cells | 200 mg qd | 90 days | Discontinue medication and receive weekly red blood cell transfusion and glucocorticoid immunosuppressive therapy | Improved | 30 days | No | - | Possible |
| 43 Damerval et al^43^ | Female  France | 82 | OC with peritoneal carcinomatosis | Pancytopenia | Leukopenia, neutropenia, and severe thrombocytopenia; Accompanied by lower limb bruising and oral bleeding; severe bone marrow hypoplasia with myelodysplastic features | 300 mg qd | 12 months | Rapid recovery of white blood cells and platelets after symptomatic treatment with blood transfusion, leukocyte elevation, and hematopoietic supportive care | Improved | 21 days | Yes, Niraparib 200 mg qd, no ADRs occurrence | Fusidic acid | Possible |
| 44 Li et al^44^ | Female  China | 59 | OC with peritoneal carcinomatosis | Thrombocytopenia | Mild decrease in whole blood cells and increase in plasma cells | 200 mg qd | 6 months | Stop medication, administer interleukin-11 and thrombopoietin subcutaneously | Improved | - | No | - | Probable |
| 45 Roxo et al^45^ | Female  Portugal | 64 | OC，  FTC with peritoneal carcinomatosis | Thrombocytopenia | Thrombocytopenia | 300 mg qd | 7 days | Discontinue medication ，high-dose dexamethasone, immunosuppression | Improved | 4 days | Yes, Niraparib 200 mg qd again, resulted in Grade 2 thrombocytopenia one week later, followed by permanent discontinuation of the medication | Levothyroxine, Mesalazine, Bisoprolol, Telmisartan, Levetiracetam | Probable |

Abbreviations: “-”, Not mentioned; PrCa, Prostate cancer; OC, Ovarian cancer; BC, Breast cancer; EC, Endometrial cancer; PaCa, Pancreatic cancer; FTC, Fallopian tube carcinoma; ILD, Interstitial lung disease; MDS, Myelodysplastic syndrome; ITP, Immune thrombocytopenia; DILI, Drug-induced liver injury; PRCA, Pure red cell aplasia; ALT,	Alanine aminotransferase; AST, Aspartate aminotransferase; DRESS, Drug reaction with eosinophilia and systemic symptoms; AATP, Acquired amegakaryocytic thrombocytopenic purpura; CTCAE, Common terminology criteria for adverse events‌; ESR, Erythrocyte sedimentation rate; CRP, C-reactive protein;

**Reference**

1.	Ishii, S.; Maekawa, S.; Amano, F.; Kikuchi, D.; Ikarashi, D.; Kato, R.; Kanehira, M.; Takata, R.; Sugimura, J.; Obara, W. Pulmonary Embolism Associated with Olaparib in BRCA2-Mutated Prostate Cancer: A Case Report *Curr. Oncol.* [Online], 2025, p. 523.

2.	Ishimoto, H.; Sakamoto, N.; Kido, T.; Ozasa, M.; Tsutsui, S.; Mori, M.; Setoguchi, D.; Takemoto, S.; Obase, Y.; Ishimatsu, Y.; Tomonaga, C.; Matsumoto, K.; Morisaki, S.; Miura, K.; Mukae, H., Drug-induced interstitial lung disease caused by olaparib: three case reports and review of the Japanese Adverse Drug Event Report database and literature. *BMC Pulm. Med.* **2023,** *23*, 289.

3.	Sun, H., Olaparib‑induced eosinophilic pneumonia(in Chinese). *Adverse Drug Reactions Journal* **2024,** *26*, 23-55.

4.	Kaitsumaru, M.; Shiota, M.; Takamatsu, D.; Blas, L.; Matsumoto, T.; Inokuchi, J.; Oda, Y.; Eto, M., Interstitial pneumonia after regression by olaparib for neuroendocrine prostate cancer with BRCA1 mutation: a case report. *Int. Canc. Conf. J.* **2023,** *12*, 131-136.

5.	Hattori, M.; Mikami, Y.; Sato, M.; Nagai, H.; Fujii, K.; Horiguchi, Y.; Shuzui, M.; Fukuda, K.; Miyamoto, Y.; Mori, M.; Hinata, M.; Kawakami, M.; Mitani, A.; Tanaka, G.; Kage, H., Olaparib-induced interstitial lung disease: A case series analysis. *Respir. Investig.* **2025,** *63*, 629-632.

6.	Si, Q.; Mao, X.; Li, W.; Yang, Q.; Ge, C., Clinical pharmacist participated in the treatment practice of a prostate cancer patient with Olaparib induced interstitial pneumonia accompanied by fever(in Chinese). *China Rational Drug Use* **2024,** *21*, 50-56.

7.	Tufoni, M.; Serena Ricci, C.; Zaccherini, G., A Case of Immune‐Mediated Liver Injury Induced by Olaparib. *Hepatology* **2018,** *68*.

8.	Zhu, K.; Drew, Y.; Jayakumar, S., Challenges in PARP inhibitor therapy: A case of Olaparib-induced liver injury and successful rechallenge with Niraparib. *Gynecol. Oncol. Rep.* **2024,** *54*, 101439.

9.	Alshelleh, M.; Park, J.; John, V.; Rishi, A.; Bernstein, D.; Roth, N., Olaparib-Induced Immune-Mediated Liver Injury. *ACG Case Rep. J.* **2022,** *9*, e00735.

10.	Kobak, S., Olaparib-related seronegative inflammatory arthritis: fırst report in the literature. *Scand. J. Rheumatol.* **2021,** *50*, 74-75.

11.	Álvarez-Salafranca, M.; Puértolas, T.; Diago, A.; Gómez-Mateo, M. C., Olaparib-associated cutaneous vasculitis in a patient with breast cancer. *J. Dtsch. Dermatol. Ges.* **2026**, e1-e4.

12.	Wheelden, M.; Cream, L.; Sivik, J.; Robson, M., A Novel Adverse Event Associated with Olaparib Therapy in a Patient with Metastatic Breast Cancer. *Case Rep. Oncol. Med.* **2018,** *2018*, 9529821.

13.	Clark, A.; Weissman, A. S.; Crowson, A. N.; Hirshburg, J., Olaparib-induced pseudoporphyria in a patient with ovarian cancer. *JAAD Case Rep.* **2023,** *39*, 58-60.

14.	Saito, M.; Fujii, K.; Banno, H.; Ito, Y.; Nakano, S., Development of Erythema Nodosum After Olaparib Treatment in a Patient With Recurrent Breast Cancer and BRCA2 Mutation: A Case Report. *Cureus* **2023,** *15*, e44864.

15.	Iinuma, S.; Takahashi, C.; Negishi, H.; Ishida-Yamamoto, A., Cutaneous arteritis during olaparib treatment for ovarian cancer. *J. Dermatol.* **2021,** *48*, e140-e141.

16.	Costa, R. E. A. R. d.; Valença, R. J. d. V., A case of hand-foot syndrome with olaparib. *Pan African Med. J.* **2022,** *43*.

17.	Maiko, F.; Ken, S.; Satoshi, Y.; Kazuki, Y.; Takashi, M.; Koji, S., Olaparib-induced cutaneous vasculitis in a patient with recurrent ovarian cancer. *Eur. J. Dermatol.* **2022,** *32*, 655-656.

18.	Sekine, M.; Terui, H.; Fujimura, T.; Asano, Y., Olaparib-Induced Purpuric Drug Eruption in a Patient with Castration-Resistant Prostate Cancer. *Case Rep. Oncol.* **2023,** *16*, 434-436.

19.	Gou, R.; Horikawa, N.; Kosaka, K., Olaparib-induced cutaneous side effects in a patient with recurrent ovarian cancer. *BMJ Case Rep.* **2022,** *15*, e249177.

20.	Stephan, C.; Magro, C., Lymphocytic and granulomatous cutaneous small-vessel vasculitis after polyadenosine diphosphate–ribose polymerase inhibitor therapy. *Clin. Dermatol.* **2025,** *43*, 762-765.

21.	Wang, Q.; Zuo, L.; Du, Q.; Ye, L.; Ruan, C.; Zhai, Q.; Xu, R., Pharmaceutical monitoring of a patient with grade IV bone marrow suppression induced by olaparib(in Chinese). *Chinese Journal of Pharmacovigilance* **2023,** *20*, 1176-1180.

22.	Wang, Y.-t.; Li, Y.-f.; Yang, J.; Chen, Z.; Zhou, H.-y.; Li, N.; Li, G.-h., Pharmaceutical care for olaparib-induced thrombocytopenia in a platinum-sensitive recurrent ovarian cancer patient(in Chinese). *Chinese Pharmaceutical Journal* **2021,** *56*, 1659-1663.

23.	Yao, H.; Liu, S., Yao HR, Liu SK. Myelodysplastic syndrome associated with olaparib(in Chinese). *Adverse Drug Reactions Journal* **2023,** *25*, 702-704.

24.	Yuan, M.; Yang, C.; Wu, Y.; Ye, M.; Luan, J., Yuan M, Yang CY, Wu YZ, et al. Olaparib induced immune thrombocytopenia(in Chinese). *Adverse Drug Reactions Journal* **2022,** *24*, 549-551.

25.	Linn, Z.; Gu, Z.; Wang, L.; Cai, S., Stem Cell Transplantation for Ovarian Cancer Patient with Associated Myelodysplasia After Maintenance Therapy with Olaparib: A Case Report. *Int. Med. Case Rep. J.* **2025,** *18*, 241-248.

26.	Dereme, J.; Asonitis, K.; Grandoni, F.; Liapi, A.; Sarivalasis, A.; Tsilimidos, G., Intersecting Anemias: PARP Inhibitor-Induced Toxicity and Autoimmune Hemolysis—A Case Report. *eJHaem* **2025,** *6*, e70112.

27.	Shammo, J. M.; Usha, L.; Richardson, K. J.; Elliott, E.; Dewdney, S.; Venugopal, P.; Cobleigh, M.; Kuzel, T. M., Olaparib-Induced Severe Folate Deficiency in a Patient With Advanced Ovarian Cancer. *J. Oncol. Practice* **2019,** *15*, 405-407.

28.	Assaf, I.; Mans, L.; Sakr, R.; Verset, G.; Van Laethem, J. L., Unusual metastasis in BRCA mutated pancreatic cancer while on maintenance Olaparib: Two case reports and review of the literature. *Eur. J. Cancer* **2021,** *157*, 63-67.

29.	Shono, M.; Murakami, K.; Ohta, M.; Nakai, H.; Matsumura, N., Interstitial lung disease caused by niraparib in ovarian cancer patient: a case report and literature review. *Jpn. J. Clin. Oncol.‌* **2024,** *54*, 352-356.

30.	Wei, Q.; Chen, D.-S.; Liu, Y.-H., Case Report: Niraparib-Related Pulmonary Embolism During the Treatment of BRCA Mutant Advanced Ovarian Cancer. *Front. Oncol.* **2022,** *12*, 853211.

31.	McLaren, A.; Cartwright, D.; Ross, E.; Roxburgh, P., First-Line PARP Inhibitors—Emerging Side Effects Require Caution: A Case of PARPi-Induced Pneumonitis. *J. Immunother Precis. Oncol.* **2021,** *4*, 171-174.

32.	Zhao, L.; Ji, C., Increase of creatine phosphokinase induced by niraparib: A case report(in Chinese). *Chin. J. Mod. Appl. Pharm.* **2024,** *41*, 3027-3028.

33.	Vázquez-Barrera, I.; Juárez-Guerrero, A.; Cuevas-Bravo, C.; Perez-Ezquerra, P. R.; Noguerado-Mellado, B., Drug Reaction with Eosinophilia and Systemic Symptoms (DRESS) caused by niraparib: a novel antineoplastic agent. *Allergy Asthma Clin. Immunol.* **2025,** *21*, 44.

34.	Badiner, N.; Carter, C.; Ioffe, Y.; Hong, L., A case of niraparib PARP-Inhibitor induced Sweet Syndrome in gynecologic cancer. *Gynecol. Oncol. Rep.* **2023,** *46*, 101162.

35.	Lei, G.; Lei, X.; Mu, J.; Zhang, Q.; Zhao, L.; YAng, L., Niraparib maintenance therapy-induced dysgeusia following surgery for ovarian clear cell carcinoma: A case report(in Chinese). *J. Cancer. Control. Treat.* **2026,** *39*, 71-74.

36.	Gabrail, N.; Smith, C., Avatrombopag Optimizes Response to Niraparib by Managing Thrombocytopenia Associated with Poly-ADP Ribose Polymerase (PARP) Inhibition in Ovarian Cancer and Breast Cancer: A Case Series. *Am. J. Case Rep.* **2020,** *21*, e927008.

37.	Jin, X.; He, Z., One case of extremely severe thrombocytopenia caused by niraparib tosilate capsules(in Chinese). *Chinese Journal of Pharmacovigilance* **2026**, 1-5.

38.	Chen, R., A case of severe thrombocytopenia induced by niraparib tosylate capsules(in Chinese). *Chin. J. Pharmacoepidemiol.* **2022,** *31*, 778-779.

39.	Ling, G.; Yili, W.; Yanhua, S.; Zhong, J.; Qu, C., A case of acquired amegakaryocytic thrombocytopenic purpura caused by niraparib(in Chinese). *J. Cancer Control Treat.* **2024,** *37*, 264-270.

40.	Xu, Y.; Yao, M.; GUo, X., A case of severe thrombocytopenia induced by niraparib(in Chinese). *Jiangsu Med. J.* **2022,** *48*, 539-540.

41.	Kobayashi, H.; Yamada, Y., Efficacy of oral ferric citrate hydrate treatment for anemia caused by niraparib: a case report. *J. Med. Case Rep.* **2022,** *16*, 440.

42.	Yücel, K. B.; Yıldız., S.; Sütcüoglu., O.; Güvercin., F. S.; Göçün., P. U.; Özdemir., N.; Yazıcı., O.; Özet., A., Niraparib-induced pure red cell aplasia. *J. Oncol. Pharm. Practice* **2024,** *30*, 210-214.

43.	Damerval, M.; Bazan, F.; Omrani, S.; Hugues, M.; Roux, M.-F.; Mayer, J., Pancytopenia in a patient treated with fusidic acid and niraparib: a case report. *Eur. J. Hosp. Pharm.* **2023,** *30*, e1.

44.	Li, J.; Yang, J.; Wang, Y., Severe thrombocytopenia induced by niraparib in ovarian cancer patients: a case report and literature review. *Front. Oncol.* **2025,** *15*, 1542646.

45.	Roxo, F.; Duarte, D.; Abreu, M. H., Immune-Mediated Thrombocytopenia Induced by Niraparib: A Clinically Relevant Case. *Cureus* **2025,** *17*, e97299.
